# Longitudinal analysis of Israeli nurses’ perceptions, trust & emotions during the COVID-19 pandemic: Shiran Bord

**DOI:** 10.1093/eurpub/ckac131.377

**Published:** 2022-10-25

**Authors:** S Bord, S Shaharabani, H Baruch, H Admi

**Affiliations:** 1 Health Systems Management Department, The Max Stern Yezreel Valley College, Yezreel Valley, Israel; 2 Economics and Management Department, The Max Stern Yezreel Valley College, Yezreel Valley, Israel; 3 Rambam, Rambam Health Care Campus, Haifa, Israel; 4 Nursing Department, The Max Stern Yezreel Valley College, Yezreel Valley, Israel

## Abstract

**Background:**

The nursing sector is the largest human resource in the healthcare system providing direct patient care. The Covid-19 pandemic has forced nursing teams into a sustained state of emergency, and with it, much uncertainty and risk taking.

**Aims:**

Examining the emotions, risk and threat perceptions, trust in the healthcare system and compliance with Ministry of Health (MOH) regulations among nurses at two points in time during the COVID-19 pandemic in Israel.

**Methods:**

Research questionnaires were distributed to nurses at the height of the pandemic’s first wave (March-May 2020). Among the respondents, 140 agreed to continue with the follow-up study. During the pandemic’s third wave (January 2021), the research questionnaire was re-sent to these respondents. Of these, 80 filled-in the second questionnaire.

**Findings:**

Naturally, there was a higher level of experience among the nursing staffs in dealing with the virus in the second as compared to the first period in time. During the first wave, approximately a fifth of the participants (21%) reported that they had cared for patients who had been confirmed as having COVID-19, while during the third wave, most of the participants (66%) reported caring for people who had been confirmed as having contracted the virus. The findings demonstrate significantly lower levels of compliance with regulations and risk perception, and a significantly higher level of emotions in the third wave as compared to the first. However, there was no change in the level of trust in the healthcare system or in the pandemic-related threat perception.

**Conclusions:**

The findings provide us with some information regarding the process the nursing staffs have undergone (and are still undergoing) in dealing with the pandemic, and may point at a ‘pandemic fatigue’ amongst them. This concept relates to progressively lower regulation compliance levels appearing over time, affected by the target population’s emotions, experiences and perceptions.

**Key messages:**

• It is important to assist the nurses’ emotional regulation with the help of professionals in the organization, form colleagues’ support groups and ensure management recognition of the situation.

• Resilience-raising steps should be taken, for example by ensuring rest times and sufficient medical and protective equipment.

